# Assessment of Knowledge about First Aid Methods, Diagnosis, and Management of Snakebite among Nursing Students: A Cross-Sectional Study from Palestine

**DOI:** 10.1155/2020/8815632

**Published:** 2020-12-16

**Authors:** Isra K. Kharusha, Suha S. Sulaiman, Ahmad M. Samara, Samah W. Al-Jabi, Sa'ed H. Zyoud

**Affiliations:** ^1^Department of Medicine, College of Medicine and Health Sciences, An-Najah National University, Nablus 44839, State of Palestine; ^2^Department of Clinical and Community Pharmacy, College of Medicine and Health Sciences, An-Najah National University, Nablus 44839, State of Palestine; ^3^Poison Control and Drug Information Center (PCDIC), College of Medicine and Health Sciences, An-Najah National University, Nablus 44839, State of Palestine; ^4^Clinical Research Center, An-Najah National University Hospital, Nablus 44839, State of Palestine

## Abstract

**Background:**

Snakebite is a serious and important medical emergency encountered in many parts of the world. The estimated number of victims of venomous snakebites in Palestine is about 100 to 150 annually, with death occurring in 2 to 3 of them. This study was designed to assess the level of knowledge on the diagnosis and management of snakebites among nursing students in Palestine, as well as their attitude towards snakebites.

**Methods:**

This was a cross-sectional survey that took place at An-Najah National University. Two hundred nursing students were asked to fill a questionnaire that was developed to assess the participants' knowledge and attitude regarding snakebite's diagnosis and management. Different knowledge scores were calculated, and the relationships between students' knowledge and their characteristics were calculated by implementing the Mann–Whitney *U* test and the Kruskal–Wallis test. The statistical significance limit of *p* values was set at 0.05.

**Results:**

The majority of the participants (57%) were fourth-year students with an average age of 20.7 ± 1. Areas of knowledge and the participants' mean scores on them were as follows: *Vipera palaestinae* snake, 5.1/13; signs and symptoms, 9.6/16; laboratory investigations, 6.1/10; anti-venom, 4.2/11; and first aid, 6.6/15. The only statistically significant differences in knowledge were between male and female students on *Vipera palaestinae* (male students scored higher, *p* value = 0.004) and between different types of residence (village dwellers scored the highest, *p* value = 0.041).

**Conclusions:**

We found knowledge gaps in many aspects of snakebite's diagnosis and management among nursing students in Palestine. Based on the results of this study, we suggest integrating more materials on this topic in the curriculum of Palestinian nursing schools, as well as more practical training, which will positively reflect on the care for snakebite victims.

## 1. Background

Snakebite is a serious and important medical emergency encountered in many parts of the world. It is a threat to public health, particularly in rural areas where snakes are more likely to be found [1]. Snakebite remains a significant source of morbidity and mortality particularly in regions where agriculture is the main occupation for a large number of people which increases the likelihood of encountering snakes [2, 3].

According to the World Health Organization (WHO), 4.5 to 5.4 million people experience snakebites per year, 1.8 to 2.7 million of which become envenomated, and the mortality is somewhere between 81,000 and 138,000 per year [4]. Despite having this high burden, snakebites are still largely neglected in the global health agenda [5]. This could be due to not attending healthcare facilities by a large proportion of the affected individuals and relying on traditional remedies instead. Some countries have a more than 70% rate of under-reporting, particularly in poorly equipped rural regions [6, 7].

Signs and symptoms of snakebites caused by different species vary widely. Three thousand different species of snakes are recognized. Five hundred species of those belong to either one of 3 families of venomous snakes which are Viperidae, Elapidae, and Hydropidae [8, 9]. Around 250 venomous snakes are listed by the WHO as medically important [10]. Snakebite is a serious medical emergency, with the result ranging from local tissue damage to the involvement of almost all vital organs of the body [5], which may lead to severe paralysis that threatens the ability to breathe, result in bleeding problems that may develop into fatal hemorrhages, and lead to permanent renal failure and extensive destruction of local tissue and possibly permanent disability or limb loss. Children tend to develop quicker and more severe reactions compared to adults due to the difference in body size. The patients of snakebite often use traditional remedies and get improper first aid measures before presenting to the hospital [8]. The mortality from snakebites is due to the lack of awareness about the proper management of victims [8]. Proper first aid in managing snakebite victims is one of the effective ways to decrease mortality.

In Palestine, an estimate of 100 to 150 cases of venomous snakebites occurs every year, 2 to 3 of whom result in death. Palestine harbors many venomous snakes, the most common of which is *Vipera palaestinae* (*V. palaestinae*) [11]. *Vipera palaestinae* is also the most dangerous venomous snake in the Middle East and accounts for the majority of venomous snakebites in Palestine [12]. Death rates from *Vipera palaestinae* envenomation approach 5% even when anti-venom is administered [13]. Despite the relatively low incidence rate of snakebites in Palestine, the high costs of treatments add to the importance of this problem for the healthcare system in Palestine, considering the economic situation and the available funds for the healthcare system in the country. The Palestinian Ministry of Health (MOH) spends about 5 million ILS (about 1.4 million USD) annually on procuring the anti-venom for the *Vipera palaestinae* venom, as reported by the Palestinian Economy Portal. The cost of the MOH per case is estimated at 24500 ILS (about 6600 USD) [14].

This study was designed to assess the levels of knowledge on the diagnosis and management regarding snakebites as well as the attitude regarding snakes and snakebites among nursing students in Palestine, who will be part of the healthcare providers in the future. The originality and importance of this study are that it explores this topic for the first time, which provides an important opportunity to improve the quality of teaching in the nursing schools in Palestine by addressing deficits in vital areas of education. Improving nursing students' education through the results of this study will in turn be reflected on patient care and the quality of health services.

## 2. Methods

### 2.1. Study Design

This study used the cross-sectional design and data was collected through a questionnaire-based interview.

### 2.2. Study Setting

The survey took place at An-Najah National University (NNU), a nongovernmental public university in Nablus, which is a city in the north of the West Bank, Palestine [15]. The nursing program at NNU is one of the largest in the country, and the choice to include students from this program was made to increase the generalizability of results from the selected students to the rest of the Palestinian nursing students. With the establishment of the Faculty of Nursing at NNU in 2004, the first university-level nursing education in the northern region of Palestine was initiated. A 4-year Bachelor of Science in Nursing (BSc) was offered, and the student must complete 138 credit hours. For a traditional four-year Bachelor of Science in the nursing program at NNU, students should predict cumulative tuition costs of at least $11,000. The following criteria (competitive acceptance) were used in order to enter the Bachelor of Science in Nursing at NNU: 1) a student with a high school certificate (i.e., Palestinian General Secondary Certificate (Tawjihi)) or equivalent, 2) the secondary school certificate's minimum grade point average (GPA) for admission in the nursing program not less than 75% or equivalent, and 3) convincing results of the personal interview in which the students background and suitability to join the nursing program are assessed [16].

### 2.3. Study Population and Sample Size

During the study period, the approximate number of nursing students at NNU was 800. A sample of 200 nursing students from NNU was selected by convenience sampling technique and interviewed in the current study. Participants were eventually chosen using a convenience sampling approach because it saves researchers time and cost and because of ease of accessibility [17].

### 2.4. Inclusion and Exclusion Criteria

In order to be included in this study, a participant was required to be a student at NNU studying in nursing school and be in one of the clinical years. Students who were still in the basic years of study or who did not complete the questionnaire were excluded.

### 2.5. Data Collection Form

We developed a questionnaire that was based on the World Health Organization (WHO) protocol for the management of snakebite injuries after reviewing the existing literature on the subject [11, 12, 18–33].

This data collection tool consisted of eight sections:

- In the first section, items on specific demographic characteristics were included. These were the age of the participant, gender, type of residence, and year of education.
- The second section contained 10 questions that address the self-evaluation of aspects related to snakebites. These aspects were the participant's self-assessment of his or her knowledge (poor, average, or good), the perceived demand for knowledge on snakebites (low, moderate, or high), any previous experience of snakebite by the participant or a relative of hers or his, any previous training on the management of snakebite the participant received, the perceived need for training on this topic, the perceived adequacy of the healthcare facilities for dealing with snakebites, the participant's source of knowledge on the management of snakebites (medical education, written media, television, the Internet, and/or family and friends), and finally the first reaction of the participant if they experience a snakebite.
- In the third section, the scale of knowledge about the *Vipera palaestinae* snake (the most common venomous snake in Palestine) was assessed, through 13 questions of the true/false type. The participant received a point for every right response, and the total score on *Vipera palaestinae* knowledge equaled the total of the points he or she scored on these 13 items.
- The fourth section investigated the participant's scale of knowledge on the symptoms and signs of venomous snakebites in general, using a list of 16 symptoms/signs of venomous snakebites. The participants were asked to identify all the symptoms/signs they thought can be caused by snakebite from that list, and the number of symptoms/signs they marked on that list was reported as the final score on symptoms and signs knowledge.
- The fifth section looked into the participant's scale of knowledge on laboratory testing that should be performed on snakebite victims, by listing 10 such tests and asking the students to identify all the laboratory tests they thought were necessary to perform on snakebite victims. The number of tests a participant selected was reported as that participant's final score on laboratory testing.
- The sixth section addressed the participant's scale of knowledge on anti-venom. It included 3 groups of items. The first group included 2 questions of the true/false type. The second group included 6 questions of the multiple-choice type, each followed by 3 possible options, as well as the option to answer with I don't know. The final group presented a list of 3 complications after the administration of anti-venom and the participants were asked to identify the potential complications after the administration of anti-venom on that list. The students received a point every time they answered correctly to a question in the first two groups, as well as one point for every complication they marked in the final group. The total of all the points they scored was the final score on anti-venom knowledge.
- In the seventh section, we tested the participant's scale of knowledge on the first aid approach to snakebites using 15 questions of the true or false type, with an option to respond with I don't know. One point was allocated for every correct response and the total of points the participant scored was reported as that participant's final score on first aid.
- The final section investigated the participant's attitude regarding snakebites and snakes generally using 8 items that address the proper attitude regarding snakebites and snakes. The students were asked to label every item as false or true, or respond with I don't know. One point was allocated for every correct answer to these items, and the total of points scored by a participant represented his or her final attitude score.

### 2.6. Validity and Reliability Study

A panel of three expert researchers who are qualified in this form of studies reviewed the content, assessed the validity of the final questionnaire, and evaluated the organization, medical terms, suitability, and completeness. After developing the questionnaire, the contents and design of the questionnaire were pilot-tested on 10 nursing students, and modifications were done as necessary. On average, conducting the interview and completing the questionnaire took around 15 minutes per student. These ten nursing students were not included in the final analysis. The questionnaire was developed in Arabic and the interviews were conducted in the same language in both the pilot study and the final study. Data for this research was collected through self-administered questionnaires. Two researchers (IK and SS) were available to answer questions and clarify any questions at all times. Reliability for internal consistency was determined using Cronbach's alpha. Cronbach's alpha coefficients were appropriate and acceptable for all scales including the scale of knowledge on *Vipera palaestinae* (0.813), the scale of knowledge on general symptoms and signs of venomous snakebite (0.871), the scale of knowledge on laboratory testing after snakebites (0.899), the scale of knowledge on use of antivenom (0.842), the scale of knowledge on first aid treatment (0.866), and the scale of attitudes towards snakebite and snakes (0.781).

### 2.7. Ethical Issue

Institutional Review Board (IRB) at NNU approved this study before it was carried out. We interviewed the students after or between their lectures. They provided verbal consent for participating in the study. The students were informed of the aims of this survey and were encouraged to give accurate and objective answers to the best of their knowledge or experience. We did not obtain any identifying information, and the data was used only for research purposes.

### 2.8. Statistical Analysis

Data analyses were conducted by IBM Statistical Package for the Social Sciences (SPSS) Version 21. Continuous variables were expressed as means and SD and categorical variables as frequencies (percentage). The Kolmogorov–Smirnov test was applied to check the normality for all scores. For variables that did not follow the normal distribution, median and interquartile ranges were used. Mann–Whitney *U* test, Kruskal–Wallis test, and Chi-square test were employed in testing the differences between different categories of variables as appropriate. *P* value was assumed to be statistically significant if it was <0.05.

## 3. Results

### 3.1. Demographic Data

A total of 200 nursing students from NNU were surveyed. The average age of the participants was 20.8 with an SD of 0.8 (years), and the majority (54%) came from rural areas. Nearly half of them (52%) were females, 42% were in their third year, and 57% were in their fourth year. Having a family member who was affected by a snakebite was not uncommon among the participants (20%), and 16% of the respondents themselves were bitten by snakes in the past. However, only 42% of students received some training in dealing with snakebites (Table 1).

### 3.2. Self-Evaluation

A minority of the students (29%) rated their knowledge of snakebites as good, and 38% believed that there was a high demand for knowledge on snakebites as shown in Table 2 which summarizes the participants' responses to self-assessment items.

### 3.3. Knowledge of Snakebites Diagnosis and Management

Table 3 shows the rates of correct responses to items on knowledge about the *Vipera palaestinae* snake. Most students (75.5%) recognized *Vipera palaestinae* as the most commonly encountered venomous snake in Palestine and 67.5% of them correctly answered that *Vipera palaestinae* toxin contains the neurotoxic compound. However, only 8.5% of the students knew that the most common sign in the clinical setting of envenomation is not rising body temperature, and 9% knew that *Vipera palaestinae* toxin contains cardiotoxin compound.

Table 4 presents the percentage of correct responses of participants about the general signs and symptoms of venomous snakebites. The majority of students knew that convulsions (80%), unconsciousness (79.5%), dizziness and vomiting (77.5%), and blurring of vision (75.5%) are among the symptoms and signs of snakebites. On the other hand, a minority of them knew that scanty or no urine output (33.5%), bleeding from gum and vomiting (35%), and dark-colored urine (35.5%) could also be symptoms and signs of snakebites.

Table 5 presents the percentages of students who appreciated the need for certain laboratory testing after snakebites. The majority of the participants knew that complete blood count (76%) and 20-minute whole blood clotting (73%) are important laboratory tests to order in snakebite cases whereas 51% knew that urine analysis must also be performed.

Table 6 shows the participants' rates of correct responses to items on knowledge about anti-venom including its preparation and the complication that may occur as a result of anti-venom administration.

The rates of the correct response to items about first aid methods in cases of snakebite are presented in Table 7. Most of the participants knew that asking the victim to stay calm is helpful (85%) and knew that the snakebite patients should be transported to the hospital as soon as possible after the bite (74.5%), whereas only 15.5% knew that applying an ice pack to the bite location is not helpful.

### 3.4. Attitudes of Students towards Snakebites and Snakes

Table 8 presents the results of the participants' attitudes regarding snakebites. Only 15.5% acknowledged that it is not necessary to kill the snake after it bites the victim, and 17% knew that it is not true that a snake would capture the image of the offender who teases it and take revenge later.

### 3.5. Comparisons between Students ' Demographic Characteristics and Their Levels of Knowledge

Table 9 presents how the knowledge score on *Vipera palaestinae* snake correlated with the students' characteristics. Knowledge score on this topic was significantly associated with gender (*p*=0.004) wherein male students scored higher than their female counterparts.

Table 10 presents the relationship between the knowledge score of symptoms and signs and the participants' characteristics. The association of knowledge score of laboratory investigations and the participating students' demographic characteristics is presented in Table 11. The relationship between the knowledge score of anti-venom and the students' characteristics is shown in Table 12.

Finally, Table 13 shows how the knowledge score of first aid correlated with the students' demographics, wherein living in a village was significantly correlated with a better knowledge score (*p* value of 0.041).

### 3.6. Comparison of the Demographic Characteristics of Students and Their Attitude Scores

Table 14 shows the relationship between attitude scores and the demographic characteristics of the participants. There was no significant association between attitude score and gender, residency, or year of study (all *p* values were >0.05).

### 3.7. Correlations between Scales

A weak yet significant positive correlation was found between *Vipera palaestinae* knowledge scale and symptoms and signs knowledge scale (*r* = 0.426, *p* < 0.001). *Vipera palaestinae* knowledge scale also had a positive correlation with laboratory testing scale (*r* = 0.279, *p* < 0.001), anti-venom scale (*r* = 0.140, *p*=0.048), first aid scale (*r* = 0.180, *p*=0.011), and attitude scale (*r* = 0.305, *p* < 0.001). Symptoms and signs knowledge scale also had a weakly positive correlation with each of the following: laboratory testing knowledge scale (*r* = 0.419, *p* < 0.001), anti-venom knowledge scale (*r* = 0.314, *p* < 0.001), first aid knowledge scale (*r* = 0.216, *p*=0.002), and attitude scale (*r* = 0.283, *p* < 0.001).

Another weakly positive yet significant correlation was found between laboratory testing knowledge scale and anti-venom knowledge scale (*r* = 0.439, *p* < 0.001), first aid knowledge scale (*r* = 0.253, *p* < 0.001), and attitude scale (*r* = 0.282, *p* < 001). We also found a weakly positive yet significant correlation between the anti-venom scale and both the first aid knowledge scale (*r* = 0.318, *p* < 0.001) and the attitude scale (*r* = 0.316, *p* < 0.001). Lastly, we found a weakly positive yet significant correlation between the first aid knowledge scale and attitude scale (*r* = 0.534, *p* < 0.001).

## 4. Discussion

Our goal in the current study was to evaluate nursing students' knowledge of snakebite diagnosis and management. We also assessed their attitude toward snakes and snakebites.

We discovered major knowledge gaps related to snakebites even though only 38% of the participating students rated the need for knowledge regarding snakebites as high, which reflects poor awareness about the importance of snakebites as a serious medical problem that causes significant morbidity and mortality. Moreover, only 43.5% of the students cited formal education as their source of information regarding snakebites, which may manifest as improper snakebite management.

This finding emphasizes the need for training classes that focus on this topic to prepare the students for properly dealing with snakebites cases.

The knowledge deficits that we found span all the field that we explored in this study as evidenced by the mean knowledge scores of *Vipera palaestinae* (5.1 out of 13), signs and symptoms (9.6 out of 16), laboratory investigations (6.1 out of 10), anti-venom (4.2 out of 11), and first aid methods (6.6 out of 15). Also, the attitude score of the participants was 3.7 out of a maximum of 8 points.

These results are in concordance with findings of studies conducted in Palestine [33], or in other countries, such as Nepal [25] and India [5], where gaps in snakebite knowledge were also reported.

### 4.1. Strength and Limitations

This was the first time that Palestinian nursing students' knowledge regarding snakebites was assessed. Considering that many cases of snakebite envenomation present to hospitals annually in Palestine, it is of high importance that all members of the healthcare team know how to deal with such cases, which was the main goal of this research. Also, this study laid the ground for further studies to evaluate the level of knowledge and practical skills of other members of the healthcare team.

One of the limitations of this study is that we assessed the participants' knowledge of dealing with snakebites in a theoretical way without addressing their practical skills, especially regarding first aid skills. Also, considering the single-center nature of this study, our results might not accurately depict the situation in all nursing schools in Palestine.

## 5. Conclusions

In conclusion, this survey revealed the gaps in the knowledge regarding dealing with cases of snakebites properly and scientifically, as well as the lack of awareness regarding how important this subject is for nursing students. We recommend focusing more on this topic considering the high likelihood that nursing students will need to deal with snakebites in their professional or even personal life. Our results also pointed to specific areas in which gaps of knowledge were excited, emphasizing the need for more studies to assess the knowledge and practices regarding first aid among other members of the healthcare teams in Palestine.

## Figures and Tables

**Table 1:** Demographic characteristics of nursing students.

Characteristics	Number (%), *N* = 200
Gender	
Male	96 (48)
Female	104 (52)

Residency	
City	72 (36)
Village	108 (54)
Palestinian refugee camp	22 (10)

Year of study	
Third year	85 (42)
Fourth year	115 (57)

**Table 2:** Nursing students' responses to self-assessment questions.

Question^*∗*^	Number (%)
“How would you rate your snakebite knowledge?”	
Good	58 (29)
Average	98 (49)
Poor	44 (22)

“How would you rate your demands for knowledge about snakebite?”	
High	76 (38)
Moderate	101 (50.5)
Low	23 (11.5)

“Have you ever experienced snakebite?”	
Yes	32 (16)
No	168 (84)

“Have any of your family members ever had a snakebite?”	
Yes	40 (20)
No	160 (80)

“Have you been getting training to deal with patients with snakebites?”	
Yes	84 (42)
No	116 (58)

“Do you believe there is a need for snakebite management training?”	
Yes	177 (88.5)
No	23 (11.5)

“Do you believe there is an appropriate facility for snakebite treatment in hospitals?”	
Yes	133 (66.5)
No	67 (33.5)

“Where did you get the snakebite knowledge?”	
Medical education	87 (43)
Television	72 (36)
Books/magazine/newspapers	42 (21)
Families/friends	33 (16.5)
Internet	99 (49.5)

“When you face a snakebite, what is your first reaction?”	
Too nervous to do anything	42 (21)
Call for a surgeon or medical colleague	132 (66)
Take simple intervention immediately	47 (23)

^*∗*^These questions were adapted from previous studies [21, 22, 33].

**Table 3:** Percentages of correct responses of nursing students to questions on knowledge regarding *Vipera palaestinae*.

Question	Number (%)
The most common venomous snake in Palestine is *Vipera palaestinae* (T)	151 (75.5)
The venom of *Vipera palaestinae* contains the neurotoxic compound (T)	134 (67.5)
Local swelling is the most common clinical symptom of envenomation (T)	105 (52.5)
Its length is 1 meter on average (T)	104 (52)
The venom of *Vipera palaestinae* contains a hemorrhagic compound (T)	95 (47.5)
The venom of *Vipera palaestinae* contains integrin inhibitors compound (T)	90 (45)
The venom of *Vipera palaestinae* contains growth factors inhibitors compound (T)	64 (32)
Males are bitten twice as often as females (T)	61 (30.5)
The mortality rate is 1.5% due to the envenomation of *Vipera palaestinae* (T)	47 (23.5)
The total level of serum cholesterol in admission can serve as an indicator of the severity of envenomation (T)	33 (16.5)
Envenomation was reported only in humans (F)	31 (15.5)
The venom of *Vipera palaestinae* contains cardiotoxin compound (T)	18 (9)
Increased body temperature is the most common clinical symptom of envenomation (F)	17 (8.5)

These questions were adapted from a previous study [33].

**Table 4:** Percentages of nursing students who recognized signs and symptoms of snakebites^*∗*^.

Sign/symptom	Number (%)
Bleeding from gum and vomiting	70 (35)
Blurring of vision	151 (75.5)
Convulsion	160 (80)
Dark-colored urine	71 (35.5)
Difficulty in respiration	149 (74.5)
Difficulty in swallowing	125 (62.5)
Dizziness and vomiting	155 (77.5)
Heaviness of eyelids	125 (62.5)
Nasal regurgitation/voice	93 (46.5)
Persistent bleeding from the bite site	80 (40)
Scanty or no urine output	67 (33.5)
Severe muscle pain	129 (64.5)
Shock/collapse	141 (70.5)
Swelling with pain and blistering	141 (70.5)
Unconsciousness	159 (79.5)
Weakness of neck muscle	114 (57)

^*∗*^Questions were adapted from a previous study [22, 33].

**Table 5:** Percentages of nursing students who acknowledged the necessity of certain laboratory tests following a snakebite.

Test	Number (%)
20-minute whole blood clotting	146 (73)
Blood grouping and Rh typing	118 (59)
Blood urea	111 (55.5)
Complete blood count	152 (76)
Creatinine	105 (52.5)
ECG	138 (69)
Electrolyte	117 (58.5)
Immunodiagnosis	131 (65.5)
Serum CPK	103 (51.5)
Urine R/E	102 (51)

These questions were adapted from a previous study [33].

**Table 6:** Percentages of correct responses of nursing to questions on knowledge regarding antivenom.

A	
Item	Number (%)
Do you think that there is a need to dilute antivenom before giving it?	67 (33.5)
Do you think that the required amount of antivenom varies with the severity of envenomation?	107 (53.5)

B	
Item	Number (%)

What is the best route to give antivenom injection?	
Intravenous (IV) (correct answer)	115 (57.5)
Intramuscular (IM)	28 (14)
Did not know	47 (23)

How many vials must at least be available if the patient has been envenomated?	
10	39 (19.5)
15 (correct answer)	26 (13)
20	10 (5)
Did not know	125 (62.5)

How many vials should be administrated initially?	
1	28 (14)
2 (correct answer)	42 (21)
4	13 (6.5)
Did not know	117 (58.5)

What do you think about the rate of infusion of each vial?	
One vial per minute	37 (18.5)
One vial per 15 minutes (correct answer)	26 (13)
One vial per 30 minutes	9 (4.5)
Did not know	128 (64)

The amount of antivenom needed for minor envenomated bites	
1-2	32 (16)
2–4 (correct answer)	29 (14.5)
5–15	6 (3)
Did not know	133 (66.5)

The amount of antivenom needed for moderate or serious envenomated bites	
1-2	19 (9.5)
2–4	20 (10)
5–15 (correct answer)	30 (15)
Did not know	131 (65.5)

C	
Item	Number (%)

Early anaphylaxis (urticaria, dyspnea, hypotension)	146 (73)
Pyrogenic reaction (fever and chill)	137 (68.5)
Diarrhea and vomiting	118 (59)

These questions were adapted from a previous study [33].

**Table 7:** Percentages of correct responses of nursing students to questions on knowledge regarding first aid in case of snakebites.

Question^*∗*^	Number (%)
“Is telling the victim to stay calm beneficial?” (yes)	170 (85)
“Should the snakebite patient be transported to the hospital soon after the bite?” (yes)	149 (74.5)
“Can envenomation be cured by antivenom therapy?” (yes)	127 (63.5)
“Should the wound of bite site be rinsed (not scrubbed) with water as soon as possible?” (yes)	120 (60)
“Are all snakebites associated with envenomation?” (no)	115 (57.5)
“Should pressure immobilization bandages be applied around the bite site?” (yes)	104 (52)
“Should healthy volunteers suck the venom out of the wound?” (no)	99 (49.5)
“Should massage of bite wound be done?” (no)	94 (47)
“Should local incisions or pricks/punctures be made over the bite site?” (no)	65 (32.5)
“Is electric current at the site of bite useful?” (no)	59 (29.5)
“Should the site of the bite be raised above the level of the person's heart?” (no)	58 (29)
“Is the application of alcohol at the site of bite beneficial?” (no)	53 (26.5)
“Should tight bands (tourniquets) be applied around the limb proximal to the bite site?” (no)	52 (26)
“Is topical instillation or application of herbs beneficial?” (no)	40 (20)
“Is the application of ice pack at the site of bite beneficial?” (no)	31 (15.5)

^*∗*^These questions were adapted from previous studies [21, 25, 33].

**Table 8:** Percentages of correct responses of nursing students to questions on attitude towards snakebite.

Attitude^*∗*^	Number (%)
“Snake venoms have medicinal value; for education, snakes are essential.” (T)	133 (66.5)
“People can suffer venom injection from an accidental scratch from the fang of a snake's severed head while handling dead snakes.” (T)	128 (64)
“Snake balances the natural ecosystem and contributes to the food chain, avoiding contamination of the soil and absorbing environmental poison.” (T)	94 (47)
“Some snakes are venomous and others are nonvenomous; before teasing, snakes do not bite.” (T)	80 (40)
“The venomous snake's head is usually oval-shaped, with regular teeth marks.” (F)	68 (34)
“Snakes have attractive patterns of appearance and movement; snakes are essential elements of biodiversity.” (T)	58 (29)
“The snake will catch the image of the victim who teases it and later take revenge.” (F)	34 (17)
“The snake should be killed as soon as possible after the victim is bitten.” (F)	31 (15.5)

^*∗*^These questions were adapted from previous studies [21, 23, 25, 33].

**Table 9:** Association between nursing students' demographic characteristics and *Vipera palaestinae* knowledge score.

Characteristic	Median ^a^[Q1-Q3]	*p* value
Gender		
Male	6 [4–8]	**0.004** ^**b, c**^
Female	5 [3–6]	

Residency		
City	4.5 [3–7]	0.158^d^
Village	6 [4–7]	
Palestinian refugee camp	5.5 [4–6]	

Year of study		
Third year	5 [3–7]	0.753^b^
Fourth year	5 [4–7]	

^a^Knowledge score regarding *Vipera palaestinae* (*V. palaestinae*) was a range within 0–13; a high score reflects more knowledge about *Vipera palaestinae*. ^b^The Mann–Whitney *U* test was used for statistical tests. ^c^The *p* value is bold where it is less than the significance level cut-off of 0.05. ^d^The Kruskal–Wallis test was used for statistical tests.

**Table 10:** Association between nursing students' demographic characteristics and signs and symptoms score.

Characteristic	Median ^a^[Q1-Q3]	*p* value
Gender		
Male	10 [7–12]	0.645^b^
Female	10 [7–13]	

Residency		
City	10 [7–13]	0.768^c^
Village	10 [7.25–12]	
Palestinian refugee camp	9 [6–12.75]	

Year of study		
Third year	10 [7–13]	0.378^b^
Fourth year	10 [7–12]	

^a^Knowledge score of signs and symptoms of snake bites was a range within 0–16; a high score reflects more knowledge about signs and symptoms. ^b^The Mann–Whitney *U* test was used for statistical tests. ^c^The Kruskal–Wallis test was used for statistical tests.

**Table 11:** Association between nursing students' demographic characteristics and laboratory investigations score.

Characteristic	Median ^a^[Q1-Q3]	*p* value
Gender		
Male	7 [4–10]	0.166^b^
Female	6 [3.25–9]	

Residency		
City	6 [3–9]	0.294^c^
Village	6.5 [4–10]	
Palestinian refugee camp	6 [4–10]	

Year of study		
Third year	6 [4–9]	0.234^c^
Fourth year	6 [4–10]	

^a^Knowledge score of laboratory investigations was a range within 0–10; a high score reflects more knowledge about laboratory investigations. ^b^The Mann–Whitney *U* test was used for statistical tests. ^c^The Kruskal–Wallis test was used for statistical tests.

**Table 12:** Association between nursing students' demographic characteristics and anti-venom score.

Characteristic	Median ^a^[Q1-Q3]	*p* value
Gender		
Male	4 [3–6]	0.969^b^
Female	4 [3–6]	

Residency		
City	4 [3–6]	0.980^c^
Village	4.5 [3–6]	
Palestinian refugee camp	4 [3–6]	

Year of study		
Third year	4 [3–6]	0.812^b^
Fourth year	4 [3–6]	

^a^Knowledge score of anti-venom was a range within 0–11; a high score reflects more knowledge about anti-venom. ^b^The Mann–Whitney *U* test was used for statistical tests. ^c^The Kruskal–Wallis test was used for statistical tests.

**Table 13:** Association between nursing students' demographic characteristics and first aid score.

Characteristic	Median ^a^[Q1-Q3]	*p* value^b^
Gender		
Male	6.5 [5–9]	0.986^c^
Female	7 [4–9]	

Residency		
City	6 [3–8]	**0.041** ^**d**^
Village	8 [5–9.75]	
Palestinian refugee camp	6.5 [4.25–8.75]	

Year of study		
Third year	7 [4.5–9]	0.755^d^
Fourth year	7 [4–9]	

^a^Knowledge score of first aid was a range within 0–15; a high score reflects more knowledge about first aid. ^b^The *p* value is bold where it is less than the significance level cut-off of 0.05. ^c^The Mann–Whitney *U* test was used for statistical tests. ^d^The Kruskal–Wallis test was used for statistical tests.

**Table 14:** Association between nursing students' demographic characteristics and attitude score.

Characteristic	Median ^a^[Q1-Q3]	*p* value
Gender		
Male	4 [3–5]	0.087^b^
Female	4 [2–5]	

Residency		
City	4 [2–5]	0.179^c^
Village	4 [3–5]	
Palestinian refugee camp	4 [2.25–5]	

Year of study		
Third year	4 [3–5]	0.324^b^
Fourth year	4 [2–5]	

^a^Attitude score was a range within 0–8; a high score reflects a higher attitude regarding snake and snake bites. ^b^The Mann–Whitney *U* test was used for statistical tests. ^c^The Kruskal–Wallis test was used for statistical tests.

## Data Availability

The datasets used and/or analyzed during the current study are available from the corresponding author on reasonable request.

## Abbreviations

WHO: World Health Organization
MOH: Ministry of Health
ILS: Israeli New Shekel
USD: United States Dollar
NNU: An-Najah National University
IRB: Institutional Review Board
SD: Standard deviation.
